# Unveiling the complex pattern of intermolecular interactions responsible for the stability of the DNA duplex†

**DOI:** 10.1039/d1sc03868k

**Published:** 2021-09-02

**Authors:** Ahmet Altun, Miquel Garcia-Ratés, Frank Neese, Giovanni Bistoni

**Affiliations:** Max-Planck-Institut für Kohlenforschung Kaiser-Wilhelm-Platz 1 D-45470 Mülheim an der Ruhr Germany giovanni.bistoni@kofo.mpg.de

## Abstract

Herein, we provide new insights into the intermolecular interactions responsible for the intrinsic stability of the duplex structure of a large portion of human B-DNA by using advanced quantum mechanical methods. Our results indicate that (i) the effect of non-neighboring bases on the inter-strand interaction is negligibly small, (ii) London dispersion effects are essential for the stability of the duplex structure, (iii) the largest contribution to the stability of the duplex structure is the Watson–Crick base pairing – consistent with previous computational investigations, (iv) the effect of stacking between adjacent bases is relatively small but still essential for the duplex structure stability and (v) there are no cooperativity effects between intra-strand stacking and inter-strand base pairing interactions. These results are consistent with atomic force microscope measurements and provide the first theoretical validation of nearest neighbor approaches for predicting thermodynamic data of arbitrary DNA sequences.

## Abstract 2

Advanced electronic structure methods provide a new insight into the intermolecular interactions responsible for the intrinsic stability of the duplex structure of human DNA.

## Graphical abstract

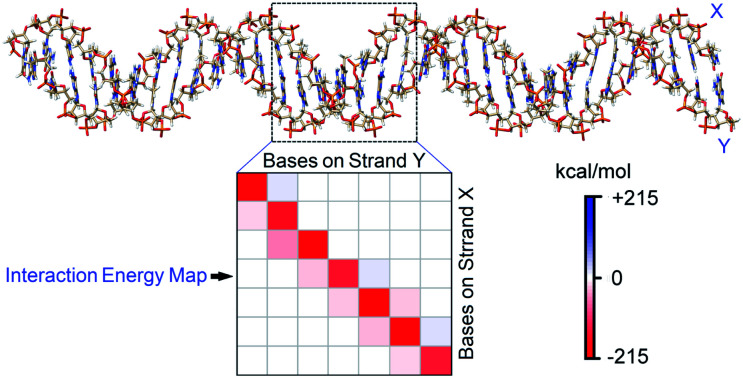

## Introduction

The double-stranded DNA structure encodes the genetic information necessary for the development and functioning of all living organisms^1^ and understanding the complex pattern of interactions responsible for the structural features of DNA is of fundamental importance in biology.

**Fig. 1:**
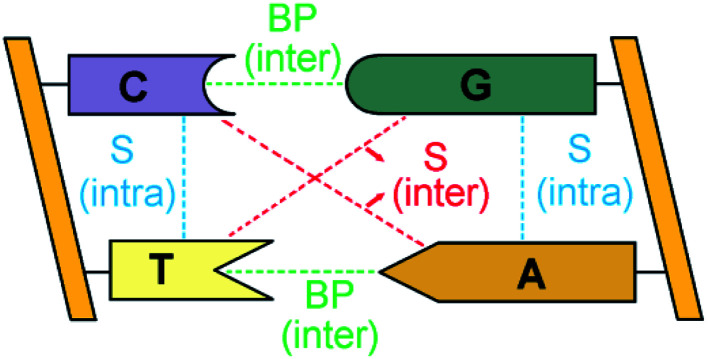
Inter-strand and intra-strand interactions of a section of double-strand DNA. BP and S denote base-pairing and stacking, respectively.

The bases of each strand of a DNA duplex lay nearly parallel on top of each other and their relative orientation is influenced by intra-strand stacking (S) interactions (Fig. 1).^2^ In addition, the two strands of DNA are held together by inter-strand S and inter-strand H-bonding interactions between Watson–Crick (WC, *i.e.*, A–T and G–C) base pairs (BPs).

In standard biology textbooks,^3,4^ inter-strand H-bonding is regarded as the major factor responsible for the stability of the DNA duplex, based on the observation that the melting temperature of DNA increases linearly with the increase of its G–C content.^5^ However, a quantitative understanding of the relative importance of base pairing *vs.* stacking interactions on the stability of the DNA duplex is still lacking.^6^ This stimulated the development of experimental probes aimed at quantifying the total stacking and the base-pairing contributions to the stability of DNA.^7–12^ These include (i) single molecule study on blunt-end DNA origami thick fibers pulled by mechanical forces;^7^ (ii) temperature-dependent polyacrylamide gel electrophoresis (PAGE) of nicked and kinked DNA molecules at different denaturating conditions;^8,9^ (iii) nano-differential scanning calorimeter (nano-DSC) and nano-isothermal titration calorimetry (nano-ITC) measurements in dilute solutions^10,11^ and (iv) stretching and unzipping of DNA for rupture force measurements under atomic force microscope (AFM).^12^ Interestingly, while mechanical studies, *i.e.*, AFM measurements, confirmed the classical textbook description by finding the rupture forces of G–C, A–T and stacking as 20, 14 and 2 piconewtons, respectively, the solution free-energy parameters derived from PAGE, nano-DSC and nano-ITC measurements^8–11^ indicate that the stability of duplex DNA arises almost entirely from stacking. Moreover, the PAGE-based stacking parameters are still consistent with the linearity of predicted DNA melting temperature on the G–C content.^8,9^ These somehow contradicting findings originate from the fact that the experimental observables that are commonly associated with the stability of DNA are influenced by a number of contributions that are difficult to disentangle experimentally, such as the concentration of the ions interacting with the backbones, temperature-dependent enthalpic and entropic effects and the intermolecular interactions between the DNA strands. In this study, rather than dissecting such contributions, we provide an in-depth, quantitative characterization of the intermolecular interactions responsible for the intrinsic stability of B-DNA at its biologically relevant duplex structure using advanced quantum mechanical (QM) methods.

Energy decomposition analysis (EDA)^13–15^ and symmetry adapted perturbation theory (SAPT)^16^ methods breakdown the QM interaction energy into physically meaningful components, and have proven instrumental in exploring such conflicting issues. However, such studies focused mostly on H-bonding and stacking interactions between just two bases^17–21^ or between two base pairs oriented at different twist angles (called base step) in the gas phase and in different dielectric media.^22–25^ The main findings of these studies can be summarized as follows: (i) the interaction between the bases in the G–C pair is significantly stronger than that in the A–T pair in the gas phase;^17–23^ (ii) due to its complex nature, there is still no consensus on the mechanism responsible for the synergistic stabilization originating from multiple H-bonds in base pairs, as discussed in a recent review paper of Guerra and coworkers;^26^ (iii) The sugar-phosphate backbone imposes geometrical constraints that destabilize base-pairing interactions^27^ and it is also essential for properly describing DNA–protein interactions, as emphasized by Hobza and coworkers;^28^ (iv) the interaction energy between base pairs or base steps decreases in solution proportional to the polarity of the solvent;^22,23^ (v) the base-pairing contribution to the stability is significantly larger than the stacking contribution, as initially demonstrated by Hessellman *et al.*^21^ and then confirmed by many subsequent computational studies^20,22,23^ (vi) inter-strand stacking is a crucial element of structural stability, especially in the GC-rich sequences.^22^ Finally, all previous computational investigations^17–23^ agree that electrostatic and London dispersion interactions are the major contribution to the base pair stability.

**Fig. 2:**
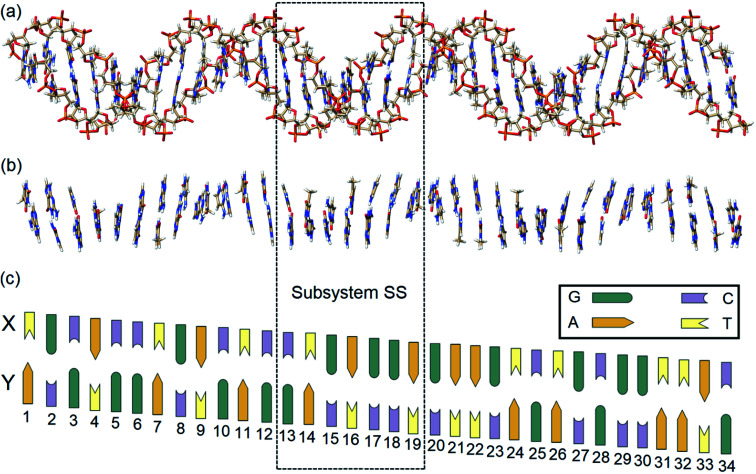
(a) Optimized structure of a real human B-DNA portion LS_BACK-C_ (2162 atoms). (b) Model system LS_N(BACK-C)_ extracted from LS_BACK-C_ by cutting out the sugar-phosphate backbones (1001 atoms). (c) Schematic DNA ladder with site numbers (*x* = 1–34). Due to the right-handed helical structure of B-DNA, the X(*x*) and Y(*x* + 1) bases are more distant than the X(*x* + 1) and Y(*x*) bases. The schematic two-dimensional DNA ladder was drawn tilted to reflect this feature. The subsystem enclosed by dashed box is labeled as SS.

In this work, state-of-the-art QM methods are used to elucidate the intermolecular interactions responsible for the intrinsic stability of human B-DNA by considering realistic DNA models of different size, including a thirty-four nucleobase-long duplex model (Fig. 2). To this aim, we apply the well-established Local Energy Decomposition (LED) scheme,^29–32^ which allows for a chemically meaningful decomposition of the interaction energy obtained at the accurate domain-based local pair natural orbital coupled cluster DLPNO-CCSD(T) level^33^ for a system containing an arbitrary number of fragments. This method has already found widespread applications in chemistry.^34–39^

In particular, our analysis relies on the recently developed Hartree–Fock plus London Dispersion (HFLD) scheme for the efficient and accurate quantification and analysis of noncovalent interaction (NCI) energies.^40^ In the HFLD scheme, the interaction between the fragments is treated at the DLPNO-CCSD level of theory, while the fragments themselves are kept at the HF level. The LED analysis is then used to single out the dispersion contribution from the other inter-fragment terms, which is then used to correct interaction energies at the HF level. On challenging benchmark sets for NCIs, this scheme provides an accuracy between that of CCSD and of the gold standard CCSD(T) method, while showing an efficiency that is comparable to that of standard mean-field approaches.^40^ Therefore, our combined HFLD/LED approach allows us to uniquely probe the nature of the interactions between all the nucleobases and the backbones in DNA at its biologically relevant structure. It is worth mentioning here that many dispersion-corrected HF approaches have been proposed,^41–47^ and semi-empirical schemes like HF-D3(BJ)^46^ or HF-3c^47^ have proven instrumental in computational studies of large biomolecular systems.^47–51^

## Computational details

Unless otherwise specified, all calculations were performed with a development version of the ORCA program package based on version 5.0.^52–54^ Very tight SCF convergence criteria were used for the isolated base pairs, while tight SCF criteria were used for all other systems. The default grid settings of ORCA 5.0, which are very conservative, were used throughout the study.

### Model systems

In order to identify and quantify the key intermolecular interactions in the DNA duplex, we defined a series of model systems of different size and charge. The initial coordinates of the thirty-four nucleobase-long duplex portion of human B-DNA (denoted hereafter as the large system LS), which is responsible for the synthesis of β-hemoglobin,^55^ were obtained using the DNA modeling server 3D-DART.^56^ The 5′-TGCACCTGACTCCTGAGGAGAAGTCTGCGGTTAC-3′ sequence (strand X) and its corresponding complementary sequence (strand Y) were considered. The 3′ and 5′ terminals of the strands were saturated with hydrogen atoms. The charged anionic phosphate groups of the sugar-phosphate backbones were kept negatively charged (total system size: 2162 atoms). The resulting coordinates were then fully optimized with the GFN2-xTB variant of the density functional tight binding method, by treating the water environment implicitly.^57^ The resulting charged model is denoted hereafter as LS_BACK-C_ (Fig. 2a). A simplified model was obtained by removing the backbones from LS_BACK-C_ and saturating the covalent bonds cut with hydrogen atoms, following the standard link atom^58^ placement protocol in ORCA. The model thus obtained (1001 atoms) is neutral and it is denoted as LS_N(BACK-C)_ (Fig. 2b).

For the sake of simplicity and unless stated otherwise, the results obtained from our extensive analyses are discussed in detail only for the subsystem SS (enclosed by a dashed box in Fig. 2), with the sequence 5′-CTGAGGA-3′. This model was built by: (i) protonating the 3′ and 5′ terminals for the subsystem extracted from LS_BACK-C_, (ii) optimizing the resulting structure at the GFN2-xTB level. The resulting model is negatively charged and it is denoted hereafter as SS_BACK-C_ (448 atoms). To assess the effect of the charge of the system on the stability of the DNA duplex, a neutral model was built by adding one hydrogen atom to one of the non-bridging oxygen atoms of each phosphate group. The resulting structure was optimized at the GFN2-xTB level and the optimized geometry is denoted as SS_BACK-N_ (462 atoms). Two simplified models were obtained by removing the backbone from SS_BACK-C_ and SS_BACK-N_ and saturating the covalent bonds cut with hydrogen atoms. The resulting SS_N(BACK-C)_ and SS_N(BACK-N)_ models feature 220 atoms. A preliminary benchmark study on smaller model systems demonstrated that the results of our analysis are essentially independent by the specific method used for the geometry optimization, as detailed in the ESI.† The HFLD/LED data obtained for all the SS and LS models are given in the ESI.†

### Calculations on isolated nucleobase dimers

In order to compare the results obtained with different computational methodologies, the interaction energies of the H-bonded WC and stacked (S) conformers of A–T and G–C pairs in the gas phase were computed using different electronic structure methods. All correlation calculations were performed with the default frozen core settings.^59^

Geometry optimizations for all conformers were carried out at the MP2 level of theory^60^ using the RIJK approach^61–63^ for the two-electron integrals in the reference calculation. The cc-pVTZ basis set was used in conjunction with its auxiliary counterparts.^64–67^

Single point DLPNO-CCSD(T)^33^ calculations were carried out using TightPNO^68^ settings. All electron pairs were included in the coupled cluster treatment. The Foster-Boys (FB)^69^ scheme was used for localizing the occupied orbitals. To approach the complete basis set (CBS) limit, two-point extrapolation was performed using the aug-cc-pVTZ and aug-cc-pVQZ basis sets,^64–66^ as described previously.^40^ Interaction energies were also corrected for the basis set superposition error (BSSE).^70^

HFLD calculations were carried out using the RIJCOSX approach^62,63,71,72^ in the SCF part. The FB scheme was employed for localizing both the occupied orbitals and the pair natural orbitals (PNOs). The default NormalPNO* settings (*T*_CutPairs_ = 10^−5^) of HFLD^40^ were used. The def2-TZVP(-f) basis set was used with its corresponding matching auxiliary basis set.^73^

Our results were compared with those obtained with HF and MP2 calculations^60^ as well as with the previous composite MP2/CCSD(T)^74,75^ and SAPT^21^ calculations. Density functional theory (DFT) calculations were also carried out, using the B3LYP^76–79^ exchange–correlation functional in conjunction with the D3(BJ)^46,80^ dispersion correction and the def2-TZVP(-f)^73^ basis set. For the large DNA models, the effect of the three-body (ABC) contribution^80^ to the D3(BJ) correction was also discussed.

### HFLD/LED analysis of the DNA duplex

DFT calculations have proven instrumental in elucidating many interesting aspects of the stability of the DNA duplex. However, different authors have emphasized the importance of benchmarking DFT results against those obtained from accurate wave function-based methods in order to test the accuracy of exchange–correlation functionals on realistic DNA models.^81,82^ In this work, our analysis relies on the HFLD scheme,^40^ which is a correlated wave function-based method that is free from any empirical parameterization. Accordingly, the dispersion interactions between the X and Y strands of the DNA were treated using conservative PNO settings, whilst intra-strand correlation effects were neglected. By combining the HFLD approach with the LED scheme,^29–31^ the HFLD interaction energy between DNA strands can be expressed as:

1 Δ*E*_int_ = Δ*E*_el-prep,X_ + Δ*E*_el-prep,Y_ + *E*_elstat(X↔Y)_ + *E*_exch(X↔Y)_ + *E*_disp(X↔Y)_

in which Δ*E*_el-prep,X_ and Δ*E*_el-prep,Y_ are the energy required to distort the electronic structure of strands X and Y, respectively, from their ground state to the one that is optimal for their interaction. Thus, they constitute the repulsive part of the inter-strand interaction. *E*_elstat(X↔Y)_ and *E*_exch(X↔Y)_ are the electrostatic and exchange interactions between the two strands, respectively. *E*_disp(X↔Y)_ represents the all-important London dispersion energy. The energy terms in eqn (1) were further decomposed into contributions corresponding to the interaction between pairs of nucleobases/backbones, by considering each base and each backbone as a separate fragment:2
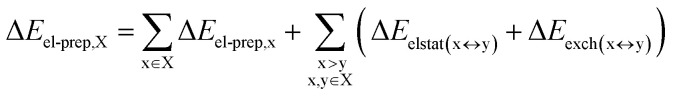
3
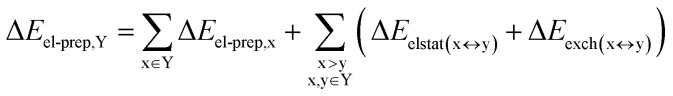
4
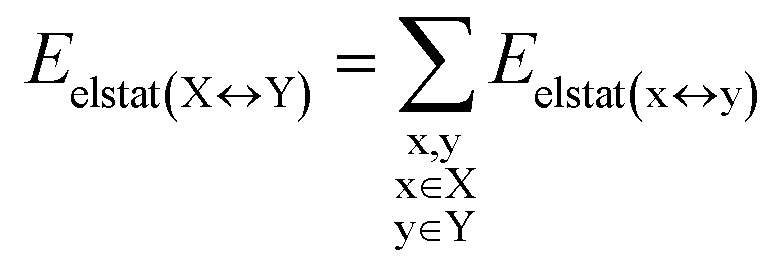
5
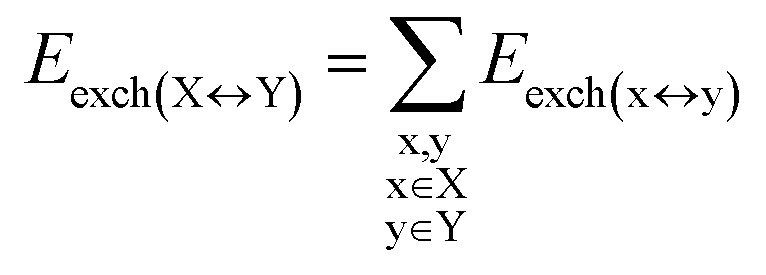
6
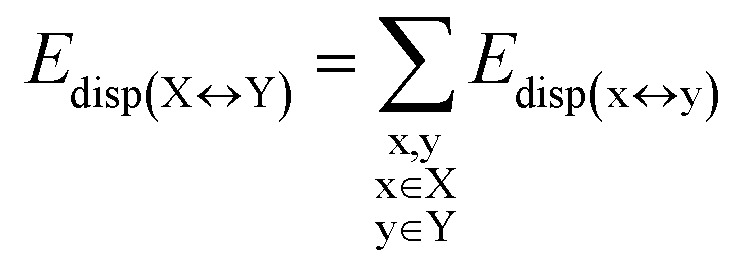
in which uppercase “X” and “Y” labels denote the strands, while lowercase “x” and “y” labels denote the individual nucleobases/backbones. Therefore, calculations on SS_BACK-C/N_ and SS_N(BACK-C/N)_ involved 16 and 14 fragments, respectively, while those on LS_N(BACK-C)_ involved 68 fragments.

For the sake of simplicity, for the model systems without the backbone (*e.g.*, LS_N(BACK-C)_), all the LED contributions were presented in the form of heat maps (the so-called LED interaction maps).^83^ The diagonal elements denote the repulsive Δ*E*_el-prep,x_ contributions associated with the individual nucleobases and backbones. Non-diagonal elements involving bases/backbones within same strand represent the changes of intra-strand electrostatic and exchange interactions upon duplex formation, *i.e.*, Δ*E*_elstat(x↔y)_ and Δ*E*_exch(x↔y)_. Non-diagonal elements involving nucleobases on different strands represent electrostatic, exchange and dispersion interactions between nucleobases on different strands, *i.e.*, *E*_elstat(x↔y)_, *E*_exch(x↔y)_ and *E*_disp(x↔y)_, respectively.

The same computational settings described in the previous subsection for isolated nucleobase dimers were used for HFLD/LED calculations on the DNA system. However, since the Δ*E*_int_ values obtained with *T*_CutPairs_ = 10^−5^ and 5 × 10^−5^ were found to be identical to each other for the SS_N(BACK-C/N)_ model, the looser *T*_CutPairs_ = 5 × 10^−5^ threshold was used for the large LS_N(BACK-C)_ calculations. The effect of water environment on the energetics was assessed using the Conductor-like Polarizable Continuum Model (CPCM),^84^ as implemented in ORCA.^85,86^ The results obtained were found to be largely independent by the specific method^87,88^ used for incorporating solvation corrections in the correlated calculations (see the ESI†).^89^ Unless otherwise specified, the results of this paper were obtained using the perturbation theory and energy PTE scheme.^87^

HFLD/LED/def2-TZVP(-f) calculations on the duplex of SS_N(BACK-C)_, SS_BACK-C_ and LS_N(BACK-C)_ require 3630, 7938 and 13 998 contracted basis functions, respectively. The corresponding computations on the DNA duplex required about 6 hours, 1.5 days and 10 days, respectively, by using four cores of a single cluster node equipped with 4 Intel Xeon CPUs. HFLD interaction energies were already shown to provide essentially converged interaction energies by using double-ζ basis sets and NormalPNO* settings on challenging benchmark sets of closed-shell adducts held together by NCIs.^40^

## Results and discussion

### Benchmark study on base pairs

Before starting our discussion on the intermolecular interactions in the DNA duplex, we tested the accuracy of the HFLD scheme on smaller systems of similar nature. The interaction energies obtained at the HFLD/def2-TZVP(-f) level of theory for the nucleobase dimers were compared with those obtained at different levels of theory as shown in Table 1.

**Table 1:** Computed interaction energies (kcal mol^−1^) of the Watson–Crick (WC) and stacked (S) conformers of nucleobase dimers in the gas phase at the HF/CBS, MP2/CBS, MP2/CCSD(T)/CBS,^74,75^ DLPNO-CCSD(T)/CBS, DFT-SAPT/CBS,^21^ HFLD/def2-TZVP(-f) and B3LYP-D3(BJ)/def-TZVP(-f) levels

	HF	MP2	MP2/CCSD(T)	DFT-SAPT	DLPNO-CCSD(T)	HFLD	B3LYP-D3(BJ)
**WC**							
A–T	−9.9	−16.9	−16.9	−15.7	−16.6	−16.2	−18.0
G–C	−24.6	−31.6	−32.1	−30.5	−31.5	−32.8	−33.2

**S**							
A–T	5.6	−15.1	−12.3	−10.9	−10.5	−11.9	−12.1
G–C	−3.4	−20.8	−19.0	−17.8	−17.7	−20.0	−19.4

For both H-bonded WC and stacked (S) conformers, HFLD results reproduce the reference DLPNO-CCSD(T)/CBS interaction energies extremely well, providing results that are also reasonably close to those obtained previously using the popular MP2/CCSD(T)/CBS^74,75^ method as well as with DFT-SAPT/CBS^21^ (Table 1). HF underestimates all the interaction energies significantly, whilst MP2 significantly overestimates those of the stacked (S) conformers. Therefore, HFLD can be considered to be a cost-effective yet accurate method for the quantification of non-covalent interactions between nucleobases.

### The role of the backbone

The inter-strand interaction energy computed for the SS_BACK-C_, SS_BACK-N_ and SS_N(BACK-C)_ models of DNA at the HF, HFLD, B3LYP-D3(BJ) and B3LYP-D3(BJ,ABC) levels in the gas phase and in water is given in Table 2. For the simplified SS_N(BACK-C)_ model in the gas phase, the DLPNO-CCSD(T_1_)/TightPNO/def2-TZVP(-f) interaction energy amounts to −177.4 kcal mol^−1^, which is very close to −178.9 kcal mol^−1^ value obtained at the HFLD/def2-TZVP(-f) level. These results provide additional evidence for the great accuracy of the HFLD method in this context. In comparison, the interaction energy obtained at the HF level of theory is significantly underestimated (−86.0 kcal mol^−1^), whilst B3LYP-D3(BJ) without and with the three-body ABC dispersion term predicts an interaction energy of −194.4 and −190.5 kcal mol^−1^, respectively. The fact that HF underestimates the inter-strand interaction with respect to DLPNO-CCSD(T), whilst B3LYP-D3(BJ) overestimates it, is consistent with the results obtained in the previous subsection for the interaction of the individual bases.

**Table 2:** Inter-strand interaction energy (kcal mol^−1^) of the DNA duplex for the subsystems SS calculated for different charge and solution states at the HF and HFLD levels, together with B3LYP that incorporates the D3(BJ) dispersion correction without and with the three-body ABC term. The def2-TZVP(-f) basis set was used in all cases

System	HF	HFLD	B3LYP-D3(BJ)	B3LYP-D3(BJ,ABC)
**Gas phase**				
SS_BACK-N_	−86.0	−185.9	−204.4	−199.0
SS_N(BACK-C)_	−86.0	−178.9^a^	−194.4	−190.5

**In water**				
SS_BACK-C_	27.4	−75.0	−104.1	−98.8
SS_BACK-N_	12.4	−88.9	−118.6	−113.2
SS_N(BACK-C)_	13.5	−80.7	−107.1	−103.2

a The corresponding DLPNO-CCSD(T_1_)/TightPNO/def2-TZVP(-f) interaction energy is −177.4 kcal mol^−1^.

In the gas phase, the interaction energy of large charged models of DNA, such as SS_BACK-C_, is known to be highly repulsive, because of the negative charge of the phosphate groups on the backbones, which leads to an insurmountable repulsive interaction in the gas phase.^90^ A common practice^91^ in QM studies of DNA for suppressing the excessive electrostatics is to artificially protonate one of the non-bridging oxygens of each phosphate group, as we have done in SS_BACK-N_. For this model, the inter-strand interaction becomes significantly attractive also in the gas phase, being −86.0, −185.9, −204.4 and −199.0 kcal mol^−1^ with HF, HFLD, B3LYP-D3(BJ) and B3LYP-D3(BJ,ABC) levels, respectively. These values are analogous to those obtained for SS_N(BACK-C)_, which indicates that, for neutral systems, the backbone provides a small contribution to the overall interaction between the DNA strands.

In addition, by incorporating the effect of the water solvent implicitly in the energetics,^86^ all models provide similar interaction energies, including SS_BACK-C_. This suggests that the interaction between the DNA and the environment counteracts the repulsion between the negatively charged DNA strands in SS_BACK-C_. Thus, in solution, the net contribution of the backbones to the interaction appears to be small, irrespective of the particular DNA model used.

It is also worth emphasizing that the inclusion of the solvent lowers the overall interaction in neutral model systems. This effect can be explained by looking at the results shown in Table 3, in which the overall solvation correction at the HFLD level is decomposed into a contribution from the CPCM dielectric, representing direct DNA-solvent interactions, plus a polarization contribution, representing how the environment influences the electronic interaction between the DNA strands (see the ESI† for a detailed discussion of how these terms are computed and for a discussion of the importance of non-electrostatic solvation corrections). The contributions from the CPCM dielectric and polarization are both very similar to each other for neutral systems with and without backbones. The contribution from the CPCM dielectric is large and positive, which causes the overall interaction to decrease in solution. In contrast, the effect of the environment on the electronic interaction between the strands is small and essentially the same irrespective of the particular DNA model employed.

**Table 3:** Decomposition of the solvation contribution to the inter-strand interaction energy (kcal mol^−1^) into DNA-solvent and DNA polarization contributions at the HFLD/def2-TZVP(-f) level of theory

	Total solvation contribution	Direct DNA-solvent contribution	DNA polarization contribution
SS_BACK-N_	97.0	123.2	−26.2
SS_N(BACK-C)_	98.1	125.0	−26.9

Importantly, for SS_BACK-N_ and SS_N(BACK-C),_ the calculated *E*_disp_ contribution to the inter-strand stability of the duplex is −99.9 and −92.9 kcal mol^−1^ in the gas phase (only ∼1 kcal mol^−1^ larger for both models in water), respectively. Therefore, the dispersion contribution to the backbone–backbone interaction is noticeable but weak compared to base–base dispersion interactions.

All these findings demonstrate that:

(i) The two DNA strands are held together by the interaction of the bases;

(ii) In solution, the net effect of the backbone to the interaction is small compared to that originating from the interaction between the bases. However, its residual contribution is likely to be very sensitive to the environment, *e.g.*, to the concentration of ions in solution. A complete theoretical characterization of temperature and ion concentration effects is beyond the scope of the present work;

(iii) London dispersion provides a fundamental contribution to the stability of the DNA duplex structure.

In the following section, we will elucidate the details of the interaction between the bases in DNA, which are responsible for the intrinsic stability of the DNA duplex. For the sake of simplicity, we will focus on the SS_N(BACK-C)_ model.

### HFLD/LED analysis of the inter-strand interaction

**Fig. 3:**
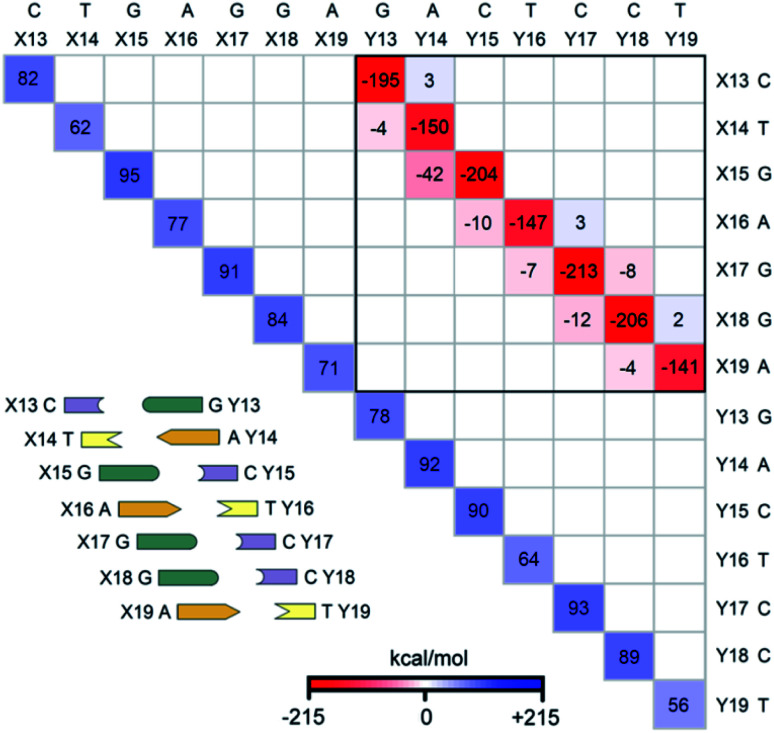
The LED interaction energy map for SS_N(BACK-C)_ in water and the corresponding schematic DNA ladder. The sum of the elements in the upper (lower) triangular submatrix, involving the interactions among bases X13–X19 (Y13–Y19), provides the overall electronic preparation energy of strand X (Y), Δ*E*_el-prep,X_ (Δ*E*_el-prep,Y_). The elements in the submatrix enclosed by solid black square denote the interaction between the bases on different strands, *i.e.*, the interactions of bases X13–X19 with bases Y13–Y19. In this submatrix, the diagonal terms correspond to the inter-strand H-bonds (base pairing), while nondiagonal terms correspond to the inter-strand stacking. Only the matrix elements greater than 2 kcal mol^−1^ in absolute values are shown on the map.

The LED interaction map provides a clear-cut visual representation of the interactions between the nucleobases and it is given in Fig. 3 for the SS_N(BACK-C)_ model system. The corresponding metadata are given in the ESI.† Note that the sum of all the elements in Fig. 3, plus the repulsive CPCM dielectric correction (direct DNA-solvent contribution in Table 3), provides the exact inter-strand interaction energy computed at the HFLD level in water, *i.e.*, −80.7 kcal mol^−1^. As discussed in the ESI,† the LED maps are only weakly affected by the specific DNA model considered or by the level of theory used for describing environmental effect.

We consider first the submatrix enclosed by a solid black square in Fig. 3, which represents the pairwise interactions between the bases on different strands, *i.e.*, the interactions of bases X13–X19 with bases Y13–Y19.

The first eye catching feature of this matrix is that the strongest inter-strand interaction is due to WC base pairing, which corresponds to the diagonal elements of this submatrix. In contrast, inter-strand stacking is effective only for the bases on neighboring sites, *i.e.*, for the X(*x*)⋯Y(*x* + 1) and X(*x* + 1)⋯Y(*x*) interactions. These results are remarkable because they provide a first theoretical validation of popular nearest neighbor (NN) models^92–100^ for predicting thermodynamic data of given DNA sequences. In fact, NN models assume no interaction between distant bases and consider only the interaction between adjacent pairs.

Moreover, our analysis also demonstrates that, due to the right-handed helical structure of B-DNA, the bases at sites X(*x* + 1) and Y(*x*) show larger overlaps than those at X(*x*) and Y(*x* + 1). Thus, the X(*x* + 1)⋯Y(*x*) stacking interactions are attractive and much stronger than the X(*x*)⋯Y(*x* + 1) stacking interactions, with the latter being usually very small or even repulsive (see the non-diagonal elements of the submatrix). We have illustrated this feature of B-DNA by plotting the schematic ladders tilted. This interesting pattern of stacking interactions is consistent with the observation that DNA sequences having the same GC-content do not necessarily have the same interaction energies,^101^ and stacking interactions among unnatural nucleobases that cannot form H-bonds are strong enough to keep the two strands together.^102^

The non-diagonal elements in the upper and lower triangular submatrices in Fig. 3 show how interactions between the base pairs on the same strand are affected by the inter-strand interaction. These numbers are essentially negligible in all cases, demonstrating that there is essentially *no cooperativity* between intra-strand stacking and inter-strand base pairing.

Finally, the diagonal elements in Fig. 3 represent the energy needed to distort the electronic structure of the bases on one DNA strand to prepare them for the interaction with the bases on the other DNA strand. They are repulsive by definition and their magnitude is slightly larger for G and C than for A and T. This effect originates from the fact that the electronic structure of G and C is perturbed by the formation of three H-bonds, whilst that of the latter by just two.

### Electrostatics, exchange and dispersion interactions

**Fig. 4:**
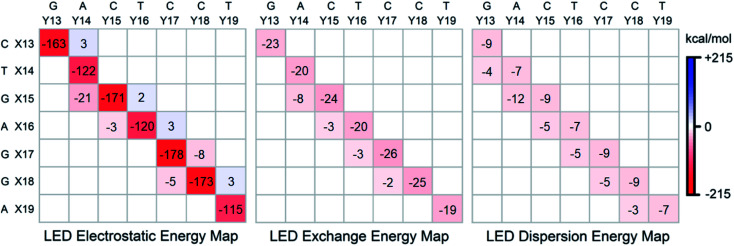
Electrostatic, exchange and dispersion energy submatrices of the LED interaction energy map for SS_N(BACK-C)_ in water.

To gain further insights into the nature of the stability of DNA duplex, the submatrix of the LED interaction energy map corresponding to the interaction between bases on different strands can be further decomposed into electrostatic, exchange and dispersion components. Such decompositions are provided for SS_N(BACK-C)_ in Fig. 4.

Consistent with previously published results on isolated dimers and base steps,^17–23^ these decompositions demonstrate that base pairing (H-bonding) is mainly of electrostatic origin also when the base pairs are in their biologically relevant structure. Exchange and dispersion also play a smaller but important role. All these attractive components are consistently larger for the G–C pair than for the A–T pair. Therefore, the stability of DNA increases with the increase of its GC-content, consistent with the above mentioned textbook explanation based on melting temperature data.^5^

As discussed above, the stabilizing effect associated with the inter-strand stacking, which is much smaller than that originating from base pairing, arises from X(*x* + 1)⋯Y(*x*) interactions. This stabilization originates from London dispersion forces to a large extent, with a smaller but noticeable contribution from the exchange interactions. The X(*x* + 1)⋯Y(*x*) inter-strand stacking interaction demonstrates some common patterns based on the size of the bases, *i.e.*, based on their overlap: it is the largest when both the X(*x* + 1) and the Y(*x*) bases are double-ringed (A or G). The interaction is still noticeably large when just one of the bases is double-ringed. However, when both of these bases are single-ringed (T or C), the X(*x* + 1)⋯Y(*x*) interaction is the smallest (even repulsive in some cases due to electrostatics), with essentially negligibly small contributions from the attractive exchange and dispersion interactions. Abbreviating the double-ringed G or A as “d”, and the single-ringed C or T as “s”, the stability sequence of the inter-strand stacking in base steps is thus sd⋯ds > dd⋯ss ≈ ss⋯dd > ds⋯sd. Finally, it is worth emphasizing here that the results just discussed remains valid irrespective of the size of the model system considered, as demonstrated in the ESI† of this work on the LS_N(BACK-C)_ model, featuring more than 1000 atoms and 13 000 basis functions.

## Conclusions

Our analysis suggests that the interaction between the two strands of large DNA models are dominated by the contribution of neighboring bases, which provides a first theoretical validation of nearest neighbor models. Consistent with previous AFM studies of large DNAs and the previous computational studies on much smaller model systems, we have found that the largest contribution to the stability of the duplex structure is the Watson–Crick base pairing, while the effect of stacking between adjacent bases is relatively small but still important for the stability of the DNA duplex. London dispersion effects were found to be essential for the stability of the duplex, while cooperativity effects between intra-strand stacking and inter-strand base pairing interactions provide a negligible contribution. To the best of our knowledge, this is the first time that a quantitative, QM-based multi-fragment energy decomposition analysis is reported for a realistic DNA model.

## Data availability

The Cartesian coordinates of all structures, the results of benchmark calculations on solvation schemes and geometries, the detailed decomposed energy terms of the inter-strand interaction energy for different B-DNA models, the corresponding heat maps, the average base step contributions and the generic references for the methods used are provided in the ESI.†

## Author contributions

G. B. devised the project, designed the computational framework and supervised the study. A. A. carried out all the calculations and wrote the original draft. G. B., A. A., M. G.-R. and F. N. contributed to the analysis of the results and to the writing of the manuscript. M. G.-R. implemented coupled-cluster solvation schemes in ORCA.

## Conflicts of interest

There are no conflicts to declare.

## Supplementary Material

SC-012-D1SC03868K-s001
